# Are normoglycaemic individuals at risk of depression? The depression-dysglycaemic phenotype from a European population-based cross-sectional study

**DOI:** 10.1186/s13690-020-00495-y

**Published:** 2020-11-07

**Authors:** Sarah Cuschieri, Julian Mamo

**Affiliations:** 1 Department of Anatomy, Faculty of Medicine and Surgery, University of Malta, Msida, MSD 2080 Malta; 2 Department of Public Health, Faculty of Medicine and Surgery, University of Malta, Msida, Malta

**Keywords:** Diabetes mellitus, Glucose tolerance, Depression, Malta, Epidemiology, Prevention

## Abstract

**Background:**

Depression is a chronic non communicable disease. It is a growing public health concern with established links with a number of co-morbidities, including diabetes mellitus. The study aimed to estimate the prevalence of depression at a population level, establish the depression sub-population phenotypic characteristics while exploring for links between depression and a spectrum of glycemic abnormalities.

**Methods:**

A nationally representative cross-sectional study was conducted in Malta between 2014 and 2016. Participants were categorized into different sub-populations according to their glycaemic status. Depression prevalence rates and phenotypic characteristics for each sub-population were established. Multiple regression analysis was performed to identify links with depression.

**Results:**

Depression was prevalent in 17.15% (CI 95%: 16.01–18.36) with a female predominance. Those with known (as opposed to newly diagnosed) diabetes had the highest depression prevalence when compared to other glycemic sub-groups. These also exhibited a significant link with self-reported depression. However, at a population level, depression was mostly prevalent within the normoglycaemic sub-population.

**Conclusions:**

The study confirms the strong link between diabetes and depression, especially, in a high risk dysglycaemic population. Of public health concern is the high depression occurrence within the normoglycaemic sub-population, which attributed for the majority of the Maltese population. In order to reduce the impact of mental health on the population, physicians may consider implementing depression screening clinical tools as part of their routine health check-ups at primary care level, irrespective of the glycaemic status of their patients.

## Background

Depression is a mental health disorder and a chronic non-communicable disease. Depression attributes to a considerable global disability and burden making this disease a public health concern [1, 2]. Across Europe the prevalence of depression has been found to vary considerably. This may be due to a number of contextual factors such as demographic, environmental, cultural and economic factors [3]. Different comorbidities such as coronary heart disease, stroke and diabetes have been found to be associated with depression [4–7]. In fact, it was reported that individuals with diabetes are two-times more likely to develop depressive symptoms when compared to none diabetics [8, 9]. However, there have been contradictory findings with regards to this depression-diabetes link [10]. Furthermore, it was reported that individuals with undiagnosed diabetes and impaired glucose metabolism are not at increased risk of depression [11].

The Mediterranean region is a unique area with similar demographic and socio-economic factors. This region was once applauded for its particular diet and the associated prevention benefits in the development of diabetes, cardiovascular disease and obesity [12, 13]. Adherence to the Mediterranean diet also has been associated with a reduced risk of depression both in the young and elderly populations [14–17]. However, the Mediterranean basin has developed an increased type 2 diabetes prevalence among other non-communicable diseases. A possible reason is the dietary patterns that have since shifted to a more Westernized pattern [18]. In fact, a number of studies across the Mediterranean region have reported this growing diabetes burden [19–21]. Hence, with the shift in dietary patterns and increase in diabetes prevalence, it is expected that the depression rate will follow suite among the Mediterranean region. Furthermore, the Northern Mediterranean countries have been reported to have industrialized characteristics while the Southern Mediterranean countries are characterized as being similar to less developed countries. The Mediterranean Islands, including the Maltese Islands found in the middle of the Mediterranean Sea, share a mixture of features of both the Northern and Southern countries including the dietary shift to a Westernized diet [22, 23]. The Maltese Islands are made up of two islands of Malta and Gozo occupying a small archipelago of 316Km2. These Islands have been well reported to feature both dysglycaemic and obesogenic characteristics. However the local depression burden at a population level has never been explored [24, 25]. These islands at the epicenter of the Mediterranean region provide a unique opportunity to explore the varying phenotypes and the relationships between the different spectrum of glycaemic status and the presence of depression at a population level. Other Mediterranean countries, both in the Northern and the Southern regions, can relate to these Islands’ characteristics. The evidence-accrued here can be useful to both public health officials and policy makers.

The aim of this study was to establish for the first time the prevalence of depression and depressive symptoms at population level, while addressing the hypothesis that a link between depression and dysglycaemia exists within the high risk dysglycaemic-obesogenic population. Dysglycaemia is a spectrum of abnormal plasma glucose levels ranging from impaired fasting plasma glucose to full-blown diabetes. The objectives included determining the biological, psychological, socio-economic characteristics of depression in accordance with the varying glycaemic status of the population. Additionally, to establish the links between groups with differing glycaemic status, depression and depressive symptoms while considering potential confounding factors including age, sex and medical co-morbidities.

## Method

### Data collection

The University of Malta conducted a nationally representative cross-sectional study between 2014 and 2016. The detailed methodology of the health examination study is found elsewhere [26]. Briefly, a single stage randomized stratified (by age, sex and locality) sample was obtained from a national register (*n* = 4000). The sample population (18 to 70 years) represented approximately 1% of each Maltese town. Those that were pregnant, were too ill to attend for the health examination or were temporarily or permanently living abroad were excluded.

Participants were invited through an invitation letter sent to their home address. Those accepting our invitation were to attend their local peripheral governmental clinic (health examination hub) during a preset weekend. The health examination hub was set up in a different town every weekend. This facilitated the attendance by bringing the hub close to each participant’s residence. An informed written consent was obtained from each participant. Trained interviewers distributed a validated demographic, social and medical co-morbidity (including self-reported depression and type 2 diabetes) questionnaire. Participants were advised to bring a list of their daily medication when they confirmed their appointment. This was listed on the questionnaire. In addition, the questionnaire also included the PHQ-9 (patient health questionnaire) as a tool for depressive symptoms [27]. During the health examination, blood pressure (repeated three times), height, weight, waist circumference and hip circumference measurements were recorded for each participant. Participants had two blood samples taken, one for fasting plasma glucose (FPG) and one for lipid profile. Ethical and data protection approvals were granted from the University of Malta Research Ethical Committee (UREC) and the Information and Data protection national commissioner respectively.

### Study population

A participation response of 47.15% was obtained (*n* = 1861). A weighting factor was applied to each participant according to the age, gender and locality in order to maintain national representativeness. The adjusted study population represented 1% of the population. The total adjusted population was of 3947 with a male predominance (*n* = 1998) and a median age of 45 years. The majority of the study population lived within the highest density populated district (Northern Harbour) and reported to be employed with the highest education level up to secondary school.

### Definitions

This study followed the PHQ-9 score definition used by Katon et al. [28], where the total score and depression severity were divided and defined as follows: 1–4 score as “No depression”; 5–14 score as “Minor depression” and 15 to 27 score as “Major depression”. Those individuals not previously aware of suffering from depression or reporting not to be on anti-depression medication but scoring > 14 in the PHQ-19 score, were considered as having *newly diagnosed depression* [29]. The segment of the study population reporting an established history of depression, were considered as being *aware of depression*. The combination of the population labelled as ‘aware of depression’ with those ‘on medication’ and ‘newly diagnosed depression’ (PHQ-9 score > 14) sub-populations were labeled as the *global depression population*.

The study population was subdivided into four glucose regulatory subgroups. These represented a continuum of glycaemic transition from normal to disordered glucose metabolism. The four sub-groups were: (1) normoglycaemia (NGR), (2) impaired fasting glucose (IFG), (3) newly diagnosed diabetes mellitus (NDM) and (4) known diabetes mellitus (KDM) subgroups. The subdivision was based on the fasting plasma glucose results obtained during the health examination survey while incorporating any self-reported history of diabetes mellitus and intake of oral hypoglycaemic agents. Those participants obtaining a fasting plasma glucose (FPG) level between 5.60–6.99 mmol/L (100.8–125.8 mg/dL) were labeled as *Impaired Fasting Glucose* (IFG) while those with a FPG > =7 mmol/L (> = 126 mg/dL) were labeled as *Newly diagnosed diabetes mellitus* (NDM), provided they were not previously diagnosed as diabetics or were on oral hypoglycaemic agents [30]. Those participants falling within the IFG criteria range were offered an oral glucose tolerance test (OGTT) two weeks after their initial examination [31]. Those undergoing the OGTT test and obtaining a 2-h glucose level beyond 11.1 mmol/L (199.8 mg/dL) were labelled also as *Newly diagnosed diabetes mellitus* (NDM), while those that remained with an FPG between 5.60–6.99 mmol/L (100.8–125.8 mg/dL) but normal 2nd hour glucose level were labeled as *Impaired Fasting Glucose* (IFG). The small proportion of individuals that fell within the “Impaired glucose tolerance” (IGT) range (i.e. 2nd hour glucose level between 7.8–11.1 mmol/L / 140.4 – 198 mg/dL) all had their corresponding FPG within the IFG range and hence for the scope of this study, were added to the IFG sub-population. Similarly, those undergoing the OGTT and obtaining a normoglycaemic profile were added to the normoglycaemic sub-population.

The participants with a previous history of diabetes mellitus or on oral hypoglycemic agents, irrespective of their measured fasting plasma glucose, were labeled as cases of *Known diabetes mellitus* (KDM). Those individuals who did not fall within these glucose dysglycaemic categories were considered to be *Normoglycaemic* (NGR). The depression prevalence rate for each sub-population was calculated.

Identifying newly diagnosed diabetics following a single fasting blood glucose reading is of common practice in population-based health examination surveys [32]. In fact, it has been reported that this case definition is used in epidemiological studies and provides a good estimate of diabetes prevalence. This is because on repeat testing, an approximate 75% of those previously tested and found to have an FPG ≥7 mmol/l had a confirmed clinical diabetes diagnosis [33, 34].

### Statistical analysis

Descriptive and analytic analyses were performed to establish the phenotypic characteristics of the depression sub-population with their corresponding glycemic status. Categorical variables were statistically analyzed using Chi squared testing while continuous variables were tested using Mann-Whitney U test since the data did not follow a normal distribution. A *p-*value of < 0.05 was considered as significant.

The most common literature based biological (sex and age), social habit (smoking), socioeconomic (education level and occupation) and medical co-morbidities (history of coronary heart disease, myocardial infarction, stroke, hypertension and anti-depressive medication) factors associated with depression were considered during the modeling analyses. Multivariate logistic regression analyses were performed while considering self-reported depression, major depression symptoms and global depression as the outcomes (respectively). The normoglycaemic sub-population was considered as the reference category.

## Results

The global depression point prevalence for the entire population was of 17.15% (CI 95%: 16.01–18.36) with a female preponderance (58.35% CI 95%: 54.59–62.00). On a national level, a total of 58,345 adults between 18 to 70 years (*n* = 340,204) have been estimated to be suffering from global depression [35]. The study’s global depression population (*n* = 677) was composed of 60.86% (CI 95%: 57.13–64.46; *n* = 412) with self-reported depression while the remainder exhibited depressive symptoms (PHQ-9 score > 14; *n* = 18) or were on medication but claimed not to be aware that they suffered from depression (*n* = 247). The latter sub-group could include those genuinely forgetful of having been once treated for depression, those who were treated for depression but they were never actually told about their diagnosis and those who used off-label anti-depression medication for other conditions (e.g. commonly Tricyclic depressants are used in cases of chronic pain) [36].

The study population (*n* = 3947) was subcategorized according to the glycaemic status. The majority of the study population had a normoglycaemic status (66.25% CI 95%: 64.76–67.71). Each glycaemic status was further categorized into the different depression categories. This was followed by estimating the prevalence for each depression status according to each glycemic category, as can be seen in Table 1. The *known diabetes* sub-group featured the highest global depression (26.01%) prevalence when compared to the other glycaemic status. Of note, this study did not identify any new depressive symptoms within the diabetic status (both KDM and NDM) sub-groups although a number of these individuals were on medication but reported not to be aware of any underlying depression. At a national level, an estimated 37,422 adults between the ages of 18 and 20 years could be normoglycaemic and have global depression (11% CI 95%: 10.06–12.01 of study population). An estimated 12,860 adults could have an impaired glucose and global depression (3.78% CI 95% 3.23–4.42), while an estimated 5613 adults could have known diabetes and global depression (1.65% CI 95%: 1.3–2.1).

**Table 1:** Distribution of the different depression categories by glycemic status

	NGR ***n*** = 2615	IFG ***n*** = 925	NDM ***n*** = 158	KDM ***n*** = 249
	[Male *n* = 1164; Female *n* = 1451]	[Male *n* = 563; Female *n* = 362]	[Male *n* = 103; Female *n* = 55]	[Male *n* = 168; Female *n* = 81]
**Depression Category**	N (%; CI 95%)	N (%; CI 95%)	N (%; CI 95%)	N (%; CI 95%)
**Self-reported**				
Total	262 (10.02%; 8.92–11.23)	91 (9.84%; 8.07–11.93)	17 (10.76%; 6.74–16.64)	42 (16.87%; 12.70–22.04)
Males	78 (6.70%; 5.40–8.29)	47 (8.35%; 6.32–10.94)	11 (10.68%; 5.91–18.28)	22 (13.10%; 8.74–19.10)
Females	184 (12.68%; 11.06–14.50)	44 (12.15%; 9.16–15.95)	6 (10.91%; 4.74–22.18)	20 (24.69%; 16.52–35.15)
**Unaware but on Rx**				
Total	159 (6.08%; 5.22–7.06)	53 (5.73%; 4.40–7.43)	12 (7.59%; 4.28–12.92)	23 (9.24%; 6.18–13.53)
Males	71 (6.10%; 4.86–7.63)	25 (4.44%; 3.00–6.50)	8 (7.77%; 3.78–14.79)	18 (10.71%; 6.81–16.38)
Females	88 (6.06%; 4.94–7.42)	28 (7.73%; 5.37–10.99)	4 (7.27%; 2.38–17.75)	5 (6.17%; 2.33–13.98)
**Unaware with PHQ-9 score > 14**				
Total	13 (0.50%; 0.28–0.86)	5 (0.54%; 0.19–1.30)	0	0
Males	2 (0.17%; 0.01–0.67)	0	0	0
Females	11 (0.76%; 0.41–1.37)	5 (0.34%; 0.12–0.83)	0	0
**Global depression**				
Total	434 (16.60%; 15.22–18.07)	149 (16.11%; 13.88–18.62)	29 (18.25%; 13.05–25.16)	65 (26.10%; 21.03–31.91)
Males	151 (12.97%; 11.16–15.03)	72 (12.79%; 10.27–15.81)	19 (18.45%; 12.06–27.11)	40 (23.81; 17.97–30.82)
Females	283 (19.50%; 17.55–21.62)	77 (21.27%; 17.36–25.79)	10 (18.18%; 9.99–30.53)	25 (30.86%; 21.83–41.63)

*Abbreviations*: *NGR* Normoglycaemiia, *IFG* Impaired fasting glocuse, *NDM* Newly diagnosed diabetes, *KDM* Known diabetes, *CI* Confidence Interval, *Rx* Treatment

The majority of the ‘global depression sub-population’ was made up of participants reporting to suffer from depression that was diagnosed by a physician. It was therefore considered as appropriate to use this sub-population to identify the phenotypic characteristics of depression at a population level, are shown in Table 2. It was observed that as the glycaemic status shifted from normoglycaemic to diabetes, the median age, the fasting plasma glucose (FPG) and the body mass index (BMI) increased. The educational levels also showed a decline (a decrease in number of education years) with this glycaemic shift. The lipid profile was observed to increase as the glycaemic status shifted from normoglycaemic to newly diagnosed diabetes. Of note, the known diabetes sub-population exhibited a better lipid profile. This follows the fact that all diagnosed diabetics are started on lipid lowering agents as part of the diabetic management regimen at the general hospital (where free medication is also authorised for all diabetics). In fact, 65% (*n* = 61) of the diabetic population with a ‘global depression’ label was on statin therapy.

**Table 2:** Phenotypic characteristics of the global depression population according to each glycaemic sub-population

Global Depression					p-value
*n*	NGR	IFG	NDM	KDM	
	434	149	29	65	
Median Age in years (IQR)	41 (27)	54 (18)	63 (8)	64 (11)	**< 0.01**
Median BMI in Kg/m2 (IQR)	26.40 (5.80)	28.90 (7.96)	28.72 (5.04)	30.86 (6.08)	**< 0.01**
Median FBG in mmol/L (IQR)	5.07 (0.48)	5.85 (0.43)	8.05 (1.91)	8.50 (3.61)	**< 0.01**
Median LDL-C in mmol/L (IQR)	3.21 (1.09)	3.32 (1.14)	3.08 (1.45)	2.41 (1.23)	**< 0.01**
Median HDL-C in mmol/L (IQR)	1.49 (0.73)	1.39 (0.54)	1.15 (0.51)	1.26 (0.37)	**< 0.01**
Median Triglycerides in mmol/L (IQR)	0.92 (0.54)	1.06 (0.99)	1.68 (0.96)	1.03 (0.57)	**< 0.01**
Median Total cholesterol in mmol/L (IQR)	5.23 (1.37)	5.50 (1.24)	5.28 (1.19)	4.38 (1.50)	**< 0.01**
Education in years					**< 0.01**
< =10 years *n* (%)	59 (13.59)	43 (28.86)	15 (51.72)	35 (53.85)	
11–13 years n (%)	270 (62.21)	78 (52.35)	10 (34.48)	26 (40)	
> =14 years n (%)	105 (24.19)	28 (18.79)	4 (13.79)	4 (2.46)	
Current smoking n (%)	187 (43.09)	38 (25.50)	14 (48.28)	21 (32.31)	0.06
Occupation					**< 0.01**
Employed n (%)	291 (67.05)	73 (48.99)	11 (37.93)	15 (23.08)	
Unemployed n (%)	9 (2.07)	5 (3.36)	0	0	
Student n (%)	18 (4.15)	11 (7.38)	0	0	
Retired n (%)	32 (7.37)	36 (24.16)	13 (44.83)	32 (49.23)	
Domestic work n (%)	84 (19.35)	24 (16.11)	5 (17.24)	18 (27.69)	
Medical history					
Coronary heart disease n (%)	25 (5.76)	6 (4.03)	8 (27.59)	14 (21.54)	**0.01**
Myocardial infarction n (%)	18 (4.15)	6 (4.03)	0	6 (9.23)	0.41
Stroke n (%)	17 (3.92)	6 (4.03)	8 (27.59)	0	0.12
Hypertension n (%)	76 (17.51)	52 (34.90)	21 (72.41)	43 (66.15)	**< 0.01**
IQR = Interquartile range					

*p*-value: Kruskal-Wallis test for Median values and Chi test for categorical variables

% representing the proportion out of the respective glycaemic status

Multivariate logistic regression analyses were performed with (i) self-reported depression, (ii) depressive symptoms cutoff score > 14 (iii) and global depression as the outcome respectively, while adjusting for sex, age, smoking, socio-economic factors and medical comorbidities. Among those with a dysglycaemic status, only those with a previously known diabetes diagnosis (KDM) were associated with a two-fold increased risk of having depression (self-reported), as seen in Table 3. The KDM sub-population also exhibited a two-fold increased risk of having ‘general depression’ although this difference held a borderline significance (*p* = 0.06).

**Table 3:** Multivariate logistic regression analyses with self-reported depression, major depression symptoms and global depression as the outcome

	Self-reported depression		PHQ-9 major depression (score > 14)		Global depression	
	Odd’s ratio (95% CI)	*p*-value	Odd’s ratio (95% CI)	*p*-value	Odd’s ratio (95% CI)	*p*-value
*Glucose status:*						
NGR	Reference		Reference		Reference	
IFG	1.05 (0.71–1.55)	0.81	1.633 (0.28–9.40)	0.58	0.82 (0.54–1.25)	0.35
NDM	0.52 (0.21–1.33)	0.18	0.87 (0.60–4.97)	1.00	0.59 (0.23–1.51)	0.27
KDM	2.36 (1.12–4.96)	**0.02**	0.95 (0.50–2.39)	1.00	2.16 (0.97–4.80)	0.06
*Adjusted Cofounding factors:*						
Female^a^	1.85 (1.26–2.71)	**< 0.01**	1.59 (0.45–5.56)	0.47	1.61 (1.07–2.43)	**0.02**
Age (years)	0.99 (0.98–1.01)	0.48	0.90 (0.85–0.95)	**< 0.01**	1.00 (0.98–1.01)	0.64
BMI (Kg/m2)	1.00 (0.97–1.03)	0.81	1.06 (0.98–1.14)	0.13	1.00 (0.97–1.03)	0.99
FBG (mmol/L)	1.03 (0.92–1.15)	0.62	1.31 (0.26–6.44)	0.74	1.03 (0.92–1.15)	0.59
LDL-C (mmol/L)	0.64 (0.18–2.24)	0.49	0.36 (0.01–18.43)	0.77	0.72 (0.23–2.29)	0.58
HDL-C (mmol/L)	0.36 (0.10–1.26)	0.11	0.27 (0.01–48.54)	0.71	0.40 (0.12–1.31)	0.13
Triglycerides (mmol/L)	0.70 (0.41–1.19)	0.19	0.49 (0.02–10.77)	0.65	0.73 (0.44–1.20)	0.21
Total cholesterol (mmol/L)	2.15 (0.63–7.34)	0.22	3.12 (0.01–44.34)	0.75	2.04 (0.66–6.33)	0.22
Education in years						
< =10 years	1.90 (1.05–3.45)	**0.04**	0.37 (0.22–13.98)	1.00	1.74 (0.93–3.29)	0.09
11–13 years	1.73 (1.08–2.77)	**0.02**	0.61 (0.44–25.88)	1.00	1.62 (0.98–2.66)	0.06
> =14 years	Reference		Reference		Reference	
Current smoking	1.99 (1.42–2.79)	**< 0.01**	1.97 (0.70–5.53)	0.20	1.99 (1.38–2.85)	**< 0.01**
Occupation						
Employed	0.71 (0.45–1.11)	0.13	0.20 (0.06–0.71)	**0.01**	0.75 (0.46–1.23)	0.26
Unemployed	1.90 (0.65–5.52)	0.24	3.72 (0.56–24.81)	0.18	1.14 (0.31–4.21)	0.84
Student	0.24 (0.05–1.11)	0.07	1.45 (0.23–7.89)	1.00	0.33 (0.07–1.58)	0.17
Retired	1.18 (0.70–1.97)	0.54	1.08 (0.18–6.64)	0.93	1.20 (0.69–2.10)	0.53
Domestic work	Reference		Reference		Reference	
Medical history						
Coronary heart disease^b^	2.90 (1.35–6.21)	**< 0.01**	1.20 (0.45–13.67)	1.00	3.43 (1.56–7.53)	**< 0.01**
Myocardial infarction^c^	0.75 (0.29–1.93)	0.55	1.78 (0.98–16.98)	1.00	0.80 (0.31–2.09)	0.65
Stroke^d^	3.03 (1.13–8.09)	**0.03**	1.29 (0.67–18.32)	1.00	3.42 (1.19–9.84)	**0.02**
Hypertension^e^	1.44 (1.00–2.06)	**0.05**	1.06 (0.33–3.36)	0.92	1.31 (0.89–1.93)	0.17
Anti-depression treatment^f^	1.12 (0.74–1.70)	0.59	0.47 (0.15–1.42)	0.18	1.38 (0.60–15.89)	1.00

*NGR* Normoglycaemia

*IFG* Impaired fasting glucose

*NDM* Newly diagnosed diabetes

*KDM* Known diabetes

^a^Male as reference

^b^ No history coronary heart disease as the reference category

^c^ No history myocardial infarction as the reference category

^d^ No history stroke history as the reference category

^e^ No history of hypertension as the reference category

^f^ No history of anti-depression treatment as the reference category

On considering the links between the potential co-founding factors within the depression sub-groups, females had an 85% increased risk of having self-reported depression (OR 1.85 CI95%: 1.26–2.71, *p* = < 0.01) and a 61% increased risk of having global depression (OR 1.61 CI95%: 1.07–2.43, *p* = 0.02). Those that smoked appeared to have a 99% increased risk of having a self-reported depression (OR 1.99 CI 95%: 1.42–2.79, *p* = < 0.01) as well as a global depression status (OR 1.99 1.38–2.85, *p* = < 0.01). As expected, a medical history of coronary heart disease, hypertension and stroke also increased the associated risk of having depression. Interestingly, a young age was associated with having severe depressive symptoms (PHQ-9 > 14; Age OR 0.90 CI 95% 0.85–0.95, *p* = < 0.01).

## Discussion

Diabetes mellitus and depression are both contributing to a growing global burden of disease, projected to increase in the upcoming years. In fact, the World Health Organization (WHO) predicted that by the year 2020, depression will rank as the second largest global burden while the International Diabetes Federation (IDF) projected a 51% diabetes prevalence increase across the world by the year 2045 [37, 38]. These projections were computed before the onset of COVID-19 pandemic. The current global pandemic has had great implications on global mental health as well as on the management and care treatment of diabetes [39, 40]. Hence, it is expected that both diabetes and depression will have a far higher global burden impact than previously projected. This study was conducted in the pre-COVID-19 era, but it is the first national study to cover both diseases. It provides essential data that can be utilized by local public health authorities as well as by other Mediterranean countries.

The established depression point prevalence in this study population was higher than the average pooled point prevalence across both European and African continents [41]. In fact, the 2017 Global Burden of Diseases (GBD) study reported that depressive disorders attributed to 6.1% of Malta’s health disability in terms of disability adjusted life years (DALYs), while diabetes attributed to 30.2% of DALYs [42]. Hence, it stands to reason that the previously known diabetes sub-population had the highest depression prevalence among all glycaemic categories, as it was previously reported by other studies [8, 43, 44]. A link was also established in this study between known diabetes and self-reported depression. This further confirms the already established link between both diseases [8, 43, 44]. Diabetes has already been established to be an attributor for the development of cardiovascular diseases [45]. This study further supports these relationships as a positive associations between depression and cardiovascular diseases were observed. Of interest known diabetes was only associated with self-reported depression and not with the presence of depressive symptoms, which coincides with the findings of a Finnish Study conducted in 2007 [10]. A probable explanation is that diagnosed diabetic individuals tend to be already engaged in a healthy lifestyle management plan including weight management as well as having a prescription for both anti-diabetic and anti-dyslipidaemic medication [30, 46]. These management plans may create stress and anxiety for the individual, resulting in depression [47]. Furthermore, it was reported that those with multi-factorial treatment plans showed higher distress levels than for those with less intensive treatments [48]. Due to the diabetic management intervention plans, these diabetic individuals are expected to have better controlled blood glucose, better lipid profile levels and more normalized body mass indices. In fact, the previously diagnosed diabetics in this study exhibit a controlled lipid profile but poor glucose and body mass control. Conversely, individuals with diabetes who reported depressive features had a poor glycaemic control and are therefore susceptible to developing other comorbidities [44, 49]. This poor glycaemic control could be related to the effect of depression on the individual’s diabetes self-care including maintaining a healthy diet, undertaking physical activity and the adherence to medication [50, 51].

Interestingly this study identified that being on anti-depression treatment was not linked with depression or depression symptoms, which contradicts the literature [10]. Although it is recommended that further research is conducted, this finding could be the result of optimal treatment resulting in a regression relationship between the disease and its symptoms. Another potential reason could be that although the PHQ-9 is an internationally validated tool [27], it has never been validated within the Maltese population. This might have led to pitfalls in its screening ability for depression symptoms within this population. Another interesting finding was the positive link between young adult age and depression symptoms, which goes against previously reported late-life and depression links [52]. However, this study’s link was only significant with depression symptoms which could be explained by the fact that young adults are typically faced by peer and media pressures which put stress on their self-esteem and confidence leading to potential depressive symptoms [53]. As one gets older, these pressures may no longer play a significant psychological impact as before. Also, there is a higher tendency for an affected person (in the older age group) to seek medical aid and starts following a psychological or medical therapy [54].

On a population level, this study observed the highest depression prevalence rate within the normoglycaemic population that makes up the majority of the total population. This finding is of public health concern, since although depression is linked with diabetes, a large proportion of the population without dysglycaemia was actually observed to have mental health issues. Low socioeconomic status, psychological stress and behavioural characteristics might be contributing to this finding, although further research is recommended. However, these findings put forward the recommendation that assessment for depressive symptoms might be considered as part of routine check-ups at primary health level, irrespective of the individual’s glycaemic status, with special attention to young adults. Clinical tools are available that can be easily implemented by physicians as part of their routine work-up [14, 31, 32]. It is recommended that a survey involving few general practitioners’ practices is conducting to reproduce this study’s findings within the Maltese population.

Increased advocacy regarding mental health should empower individuals across all ages to seek medical and psychological help if the need arises. In fact, recently “A mental health strategy 2020- 2030” has been implemented in Malta in order to reduce the burden and impact of such disease on the population [55].

### Strengths and limitations

This is the first study conducted at a population level within Malta, targeting depression and dysglycaemia. The study protocol was based on the European Health Examination protocol and hence comparative analyses could be performed.

A number of limitations were present. A response rate of 47.15% was obtained which was in keeping with a previous pilot Maltese health examination survey and other health examination surveys across Europe [56, 57]. In recent years it has been noted that a steep decline in participation rates is occurring due to the increase in the amount of available epidemiological surveys and invasive procedures [58]. Hence, the respondent data has been weighted to reflect the origin sample population thereby preserving representation.

The depression data was self-reported, so that human recall error and bias may have been present. Depression symptoms were determined using the PHQ-9 tool, which is a self-reported tool that may miss less severe cases of depression [33]. For the purposes of this study, only major depressive symptoms were considered. This might have had an effect on the overall findings. Additionally, this tool was never validated for the Maltese population, hence screening results may not have been optimal. This study was based on cross-sectional data and subject to information bias. The establishment of chronological links between cause and effects is not possible. The glycaemic status was based on one single fasting plasma glucose reading and medical history. Only those with IFG were offered an OGTT. Since an OGTT was not offered as the baseline test to everyone, a proportion of dysglycaemic individuals might have been missed and therefore this acts as a limitation.

## Conclusion

The study confirms the strong link between diabetes and depression in a high risk dysglycaemic population. However, of public health importance is the high depression occurrence within the normoglycaemic sub-population which make up the majority of the total Maltese population. In order to reduce the impact of mental health on the population, physicians should consider implementing depression screening clinical tools as part of their routine health check-ups, irrespective of the glycaemic status of their patients.

## Data Availability

All data generated or analysed during this study are included in this published article.

## Abbreviations

BMI: Body mass index
CI: Confidence interval
DALYs: Disability adjusted life years
FPG: Fasting plasma glucose
IDF: International Diabetes Federation
IFG: Impaired fasting glucose
IGT: Impaired glucose tolerance
IQR: Interquartile range
GBD: Global Burden of Disease
KDM: Known diabetes mellitus
NDM: Newly diagnosed diabetes mellitus
NGR: Normoglycaemia
OGTT: Oral glucose tolerance test
OR: Odds ratio
PHQ-9: Patient health questionnaire
WHO: World Health Organization
